# Unleashing recyclates for enhanced mechanical and durability performances of ternary blended concrete

**DOI:** 10.1371/journal.pone.0336231

**Published:** 2025-11-05

**Authors:** Solomon Oyebisi, Mohammed Alquraish

**Affiliations:** 1 Civil Engineering Department, The University of South Pacific, Suva, Fiji Islands; 2 Industrial Engineering Department, University of Bisha, Bisha, Saudi Arabia; SASTRA Deemed University, INDIA

## Abstract

The recycling of waste materials as environmentally friendly cement alternatives to lessen the impact of CO_2_ emissions and safeguard natural resources associated with cement manufacturing cannot be over-emphasized. This study investigates the effects of recycled waste materials such as shea nutshell ash and ground oyster seashell as Portland limestone cement substitutes on the mechanical, durability, and sustainability performances of ternary blended concrete. Shea nutshell ash and ground oyster seashell were partially used as cement replacement at 5–15 wt. % and tested for mechanical properties after 3–120 curing ages. Chemical resistances and drying shrinkage were conducted as durability performance after 120 and 1–120 curing ages. The results revealed higher compressive, flexural, and split tensile strengths at later ages, with about a 3% increase at 10 wt. % substitution after 90 curing ages than the control concrete. Ternary blended concrete samples, at 5–10 wt. % of shea nutshell ash and ground oyster seashell replacement levels, resulted in improved acidic, sulfate, and drying shrinkage resistances by 11–40%, 12–53%, and 9–34%, compared to the control samples. Ultimately, this research recommends an optimum of 10 wt. % shea nutshell ash and ground oyster seashell as cement alternatives, enhancing mechanical durability properties of ternary blended concrete.

## 1. Introduction

The exponential rise in urbanization and industrialization has unquestionably increased the demand for Portland cement (PC). Due to this spike, more cement clinker is produced, which uses a lot of energy and emits carbon dioxide (CO_2_) into the atmosphere [1]. About 23% of the world’s CO_2_ emissions come from the construction sector alone [2]. Since 1970, there has been a steady increase in both global cement output and consumption, following a constant annual growth pattern [3]. Cement is a frequently used building material due to its stability, adaptability in the design of building structures, inherent fire resistance, high compressive strength, and minimal maintenance requirements [4]. On the other hand, producing cement clinker requires a lot of energy. The primary source of CO_2_ emissions to the atmosphere is typically attributed to the production of PC, which is necessary to build concrete. PC production accounts for 2–3% of global energy consumption, while the usage of concrete contributes significantly to greenhouse gas emissions, accounting for 5–8% of global emissions [5]. The construction sector needs to adapt to the current climate and look for alternatives to PC, infamous for requiring high energy and emitting CO_2_ into the atmosphere [3]. Strategically using supplementary cementitious materials (SCMs) as cement alternatives is one of the ways to address these issues [6,7]. For many years, solid waste has been recycled into cement-based materials. It dramatically reduces the waste that would otherwise be disposed of and released into the environment through landfills. Additionally, it encourages sustainable growth by using waste in place of cementitious materials. For instance, innovative and ecologically beneficial alternative materials such as shea nutshell [8] and ground oyster seashells (GOS) [9,10] are recycled for concrete production. The importance of concrete will keep increasing as new uses and environmentally friendly technologies are developed [11]. More than 18 billion tons of concrete are expected to be needed annually by 2050 [12]. Thus, incorporating SCMs into concrete production promotes sustainable development and production [6].

In 2022, the global production of sheanuts was estimated to be 815825.10 metric tons [13]. Shea butter is made from shea nuts, whereas shea nutshell ash (SNA) is formed by calcining a shea nutshell [14,15]. One cannot overstate the shea tree’s multifarious applications, particularly its extremely valuable butter product used in several vital businesses. Nevertheless, the residue left over from the shea butter extraction, which is carelessly discarded, presents problems for the environment [16]. Due to this waste’s proven high-energy value, 5420 metric tons are exported to the United Kingdom each year to be co-combusted in coal power plants [16]. At worst, this solid residue is typically landfilled, where it eventually breaks down into organic manure over an extended period. Consequently, recycling shea nutshells as a cement alternative is crucial. A relevant investigation found that replacing 15% of the PC with SNA was sufficient to achieve the 28-day target compressive strength [8]. To stabilize earth blocks, a mixture of 2 wt. % SNA and 4 wt. % PC is appropriate [17]. In place of PC and fine aggregates, 15 and 30 wt. % of kaolin clay and shea nutshell particles were added to the concrete, improving its strength, durability, and physical properties [18]. Shea nutshells were shown to be suitable materials that can be recycled to improve the economics of clay brick manufacture in several characterization experiments [16].

Undeniably, there is a significant and growing amount of waste seashells [19]. Global production of waste seashells amounts to roughly 45,000 tons annually [20]. There are 370–700 g of leftovers (primarily shells) for every 1 kg of oysters [19]. There are numerous varieties of available waste seashells, including cockle, periwinkle, scallop, oyster, and mussel shells [19]. Over time, seashells have been utilized for a wide variety of applications, including building materials, artistic and architectural embellishments, musical instruments, and resources for medicine and pharmacy [21]. Generally, seashells possess high calcium content, which improves the concrete’s mechanical and physical characteristics [19]. The effects of 5–30 wt. % of GOS, mussel shell ash (MSA), and scallop shell ash (SSA) on the mechanical, pore structure, fresh, and thermal properties of lightweight foamed concrete were investigated. A 5 wt. % bivalve clam seashell ash (BCSA) [20] and 4 wt. % of ground cockle seashells (GCS) [22] showed the best replacement level with cement for improved mechanical and durability properties. An extensive analysis of recycled seashells demonstrated improved strength at 5–15 wt. % substitution of seashell powder with cement [23,24].

From the literature perspective, ternary blended concrete (TBC) containing SCMs is currently garnering interest globally due to its capacity to improve blended concrete’s mechanical, physical, and durability properties while lowering costs and adverse environmental effects [25–27]. The utilization of several SCMs has gained attention due to the effectiveness of mixing more than two different types of additives with PC. TBC achieves a high cement substitute, enabling the production of sustainable and ecologically friendly concrete [28]. Ternary blends are essential for maintaining the excellent performance of concrete while reducing the cement content. Ternary blends have demonstrated that the shortcomings of a binary blend can be addressed by utilizing two or more SCMs. Thus, ternary mix systems can maintain high levels of strength and durability while improving efficiency and environmental performance compared to binary mix systems [29,30]. TBC increases compressive strength over time by exhibiting a synergistic effect on all mixes [31]. For example, the TBC with 15% rice husk ash (RHA) shows a notable 24% increase in compressive strength and a 3.5% decrease in CO_2_ emissions [26]. When cured at both normal and high temperatures, ternary mixes incorporating 5–8% quartz filler (QF) and silica fume (SF) and SF and limestone powder (LSP) showed significant improvements in mechanical and durability characteristics [32]. On the other hand, adding SCMs to concrete and mortar production is a popular way to increase their durability in harsh environments [33–35]. For example, incorporating silica fume (SF), limestone powder (LP), natural pozzolan (NP), or ground blast furnace slag (GBFS) enhances the resistance to sulfuric acid. It improves the mechanical properties of blended cement mortars [33]. Additionally, including NP lessens the penetration of sulfate and chloride ions of blended concrete and increases its resistance to hydrochloric and sulfuric acids [34,36]. Despite the possibilities of using SNA and GOS as binary substitutes for cement, research on the synergistic effects of SNA and GOS as cement substitutes in ternary blended concrete production is lacking. This gives novelty to the present study and fills the gaps of existing studies. Moreover, no research has examined the degradation of cement-based SNA-GOS-modified concrete submerged in harsh environments, such as MgSO_4_ and H_2_SO_4_ solutions that mimic the milieus above. This also justifies the conduct of this study.

This research investigates the mechanical and durability properties of TBC incorporating SNA and GOS as cement alternatives. Cement was partially replaced with SNA and GOS at 5–15 wt. % and the mix design used concrete strength classes 25 and 30 MPa. Mechanical properties were tested after 3–120 days of curing. Following a 120-day immersion in 5% H_2_SO_4_ and 5% MgSO_4_ solutions, TBC samples were evaluated for residual weight and strength (durability properties). The TBC specimens were tested for drying shrinkage after 1–120 curing ages and compared to the control specimen. TBC samples were characterized for microstructures and elemental compositions, mineralogical identification, and thermal decomposition and phase transitions using a scanning electron microscope-energy dispersive X-ray spectroscopy (SEM-EDX), X-ray diffractometer (XRD), and thermogravimetric analysis (TGA)/differential thermal analysis (DTA) and differential scanning calorimetry (DSC). After obtaining shea nuts and oysters, their shells are indiscriminately discarded, significantly impacting the environment, leading to air, water, and soil contamination, along with potential health problems and ecological damage. Besides, improper waste disposal releases harmful chemicals, contributes to greenhouse gas emissions, and disrupts natural ecosystems. Thus, recycling shea nutshells and oyster seashells as SNA and GOS helps minimize landfill and disposal problems. The findings presented herein would be helpful in determining the crucial factors that could impact the mechanical and durability prospects of cement-based concrete modified with SNA and GOS. The research would significantly improve concrete’s functional properties while lowering the cement content required to make concrete and promoting sustainable concrete production.

## 2. Materials and methods

### 2.1. Materials

**Fig 1:**
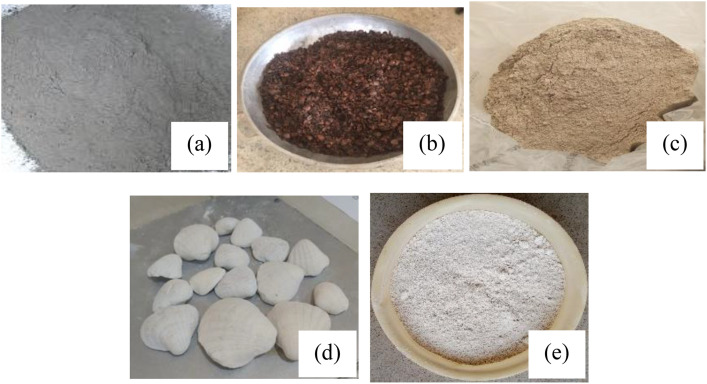
Binding materials used (a) PLC, (b) Shea nutshells, (c) SNA, (d) Oyster seashells, and (e) GOS.

**Fig 2:**
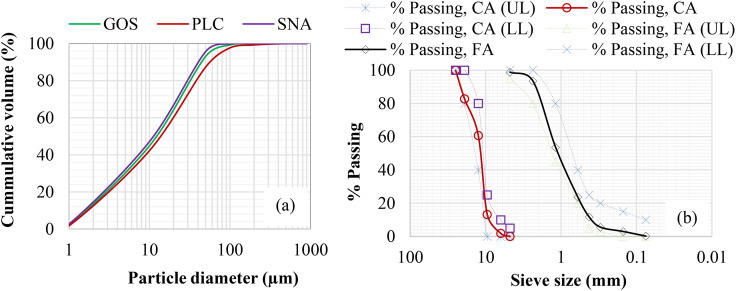
PSD for (a) binders and (b) aggregates.

All binding material, such as Portland limestone cement (PLC, 42.5 R), shea nutshells, and oyster seashells, were obtained locally and sun-dried for 7 days to facilitate the recycling operations. Under a controlled setting, shea nutshells were calcined at 700 °C for 3 h, yielding roughly 30 wt. % SNA, as seen in Fig 1. The oyster seashells were thoroughly cleaned in water to get rid of any remaining sand or salt. To reduce any leftover organic content, the shells were immersed in a vinegar-water bath for 24 h [20]. After 24 h, the shells were heated to 105 °C for 2 h to achieve a dehydrated state [20]. The prepared seashells were pulverized for 1 h at a 1000 rev/minute speed using an abrasion machine. Subsequently, the produced particles were filtered using a 45-µm BS sieve to yield the GOS depicted in Fig 1. Granite and river sand, having 12.5 and 4.75 mm particle sizes and satisfying BS requirements, were used as coarse aggregates (CA) and fine aggregates (FA) [37]. Binding materials were evaluated for specific gravity (SG), specific surface area (SSA), and bulk density (BD) per BS [38]. Table 1 shows the physical and chemical properties of the materials used. The chemical compositions were analyzed by an XRF analyzer (JOEL-JSM 7600F). The particle size distribution (PSD) of the binding materials, as ascertained by laser diffraction using a Beckman Coulter LS-100 model, is displayed in Fig 2 (a). The aggregate grades, according to the BS [37], are shown in Fig 2 (b), together with the lower limits (LL) and upper limits (UL).

**Table 1:** Chemical and physical properties of materials used.

Chemical compositions					
**Oxide content (%)**	**PLC**	**GOS**	**SNA**	**FA**	**CA**
CaO	64.90	81.62	6.62	**–**	**–**
SiO_2_	21.60	6.99	54.85	**–**	**–**
Al_2_O_3_	5.85	2.43	18.78	**–**	**–**
Fe_2_O_3_	2.78	2.40	8.10	**–**	**–**
MgO	1.42	3.05	1.26	**–**	**–**
Na_2_O	0.14	0.02	0.75	**–**	**–**
K_2_O	0.19	0.17	1.85	**–**	**–**
SO_3_	2.03	6.99	1.15	**–**	**–**
P_2_O_5_	–	0.55	0.25	**–**	**–**
Ti_2_O	–	0.15	1.38	**–**	**–**
LOI @ 800 °C	1.38	0.75	3.75	**–**	**–**
**Physical properties**					
SG	3.15	2.45	2.25	2.60	2.66
BD (kg/m^3^)	1440	1005	998	1620	1650
SSA (cm^2^/g) Blaine	3750	4250	4950	**–**	**–**
Water absorption (%)	**–**	**–**	**–**	0.30	0.20
Moisture content (%)	**–**	**–**	**–**	0.70	0.80

Table 2 demonstrates that GOS and SNA met the ASTM pozzolanic requirements [39], where the total amount of silica (SiO_2_), alumina (Al_2_O_3_), and ferrite (Fe_2_O_3_) was greater than 50%, and the LOI was less than 6%. Class C Pozzolan is assigned to GOS because its CaO concentration is greater than 18%; Class F Pozzolan is assigned to SNA because its CaO level is lower than 18%.

**Table 2:** Mix proportions (kg/m^3^).

Grade	Mix ID	Binder			FA	CA	Water	Water-to-binder ratio
		PLC	SNA	GOS				
C25 MPa	TB0 (100 PLC) wt. %, control sample	326	0	0	862	919	199	0.61
	TB5 (90 PLC + 5 SNA + 5 GOS) wt. %	293	16.50	16.50	858	919	199	
	TB10 (80 PLC + 10 SNA + 10 GOS) wt. %	261	32.50	32.50	852	919	199	
	TB15 (70 PLC + 15 SNA + 15 GOS) wt. %	228	49	49	842	919	199	
C30 MPa	TB0 (100 PLC) wt. %, control sample	369	0	0	828	919	199	
	TB5 (90 PLC + 5 SNA + 5 GOS) wt. %	332	18.50	18.50	822	919	199	
	TB10 (80 PLC + 10 SNA + 10 GOS) wt. %	295	37	37	814	919	199	
	TB15 (70 PLC + 15 SNA + 15 GOS) wt. %	258	55.50	55.50	807	919	199	

### 2.2. Experimental methods

#### 2.2.1. Design of mix proportions.

This study used the ACI standard to proportion the concrete specimens [40], having 25 and 30 MPa specified compressive strengths and 0.61 and 0.54 water-to-binder ratios to achieve 25 and 50 mm slump values [40]. A binary blend of SNA or recycled seashells with cement at 15 wt. % optimum improved the mechanical properties of blended cement concrete [8,23]. Thus, each of SNA and GOS was replaced by 5–15 wt. % of PLC to benchmark the performance characteristics of ternary blended concrete. The details are presented in Table 2. The control sample is denoted by TB0. The TBC samples are denoted by TB5, TB10, and TB15, having a synergistic combination of SNA and GOS at 5, 10, and 15 wt. % each. Three samples were made for each proportion, and the mean value of the three specimens was used for the analysis.

#### 2.2.2. Workability and mechanical properties.

**Fig 3:**
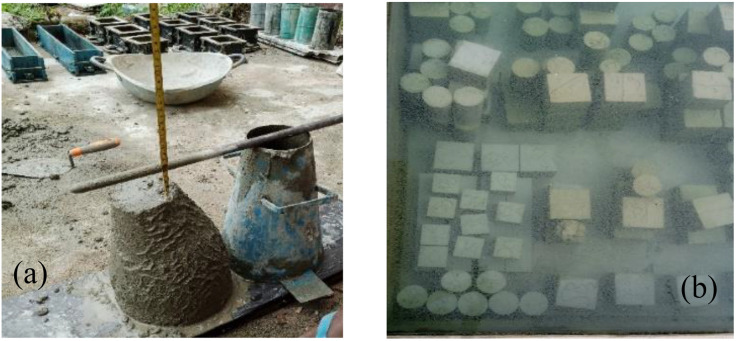
Experimental operations showing (a) the slump test and (b) the curing of concrete samples.

**Fig 4:**
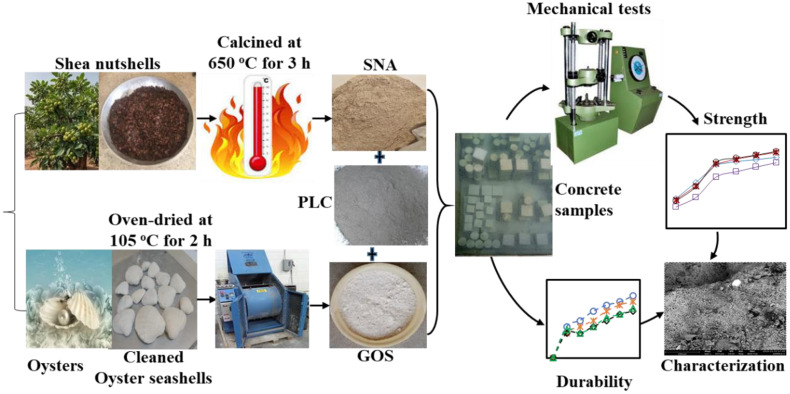
Research methodology’s flowchart.

Setting time tests were conducted using the Vicat apparatus per BS ISO [41]. The slump test was used to assess the consistency of fresh TBC samples. The test used a standard cone with internal dimensions of 200 mm base diameter, 300 mm height, and 100 mm top diameter per BS standards [42]. Concrete mixes are prepared and tested per the BS’s procedure [43]. The compressive, flexural, and split tensile strengths were tested on 100 mm × 100 mm × 100 mm cubes [44], 500 mm × 100 mm × 100 mm prismatic beams [45], and 200 mm × 100 mm cylinders [46]. All concrete samples were cured by water immersion at 23 ± 5 °C and 65 ± 5% RH [47] and tested after 3, 7, 28, 60, 90, and 120 days. Figs 3 and 4 present the experimental operations and the research methodology’s flowchart.

#### 2.2.3. Strength prediction and modelling.

This research developed models the relationship between compressive strength (f_cu_) and flexural strength (f_r_), and compressive strength (f_cu_) and split-tensile strength (f_ct_). The models were validated with previous models, as presented in Table 3, to verify the models’ accuracy and applicability.

**Table 3:** Model development.

S/N	Relationship	Model	Reference
1	f_r_ and f_cu_	f_r_ = 0.60(f_cu_)^0.50^	[48]
		f_r_ = 0.40(f_cu_)^0.70^	[49]
		f_r_ = 0.245(f_cu_)^0.823^	[50]
2	f_ct_ and f_cu_	f_ct_ = 0.36(f_cu_)^0.50^	[48]
		f_ct_ = 0.30(f_cu_)^0.67^	[49]
		f_ct_ = 0.208(f_cu_)^0.764^	[50]

#### 2.2.4. Durability.

(a) **Chemical resistance**

Concrete’s durability was examined to determine its resilience to chemical attacks. Three cubes measuring 100 mm × 100 mm × 100 mm were cast for every TBC sample to conduct the test. After that, the concrete cubes were subjected to 5% MgSO_4_ and 5% H_2_SO_4_ solutions for 120 days to test for sulfate and acidic attacks. The solutions were made by adding 5% MgSO_4_ and 5% H_2_SO_4_ by water weight to achieve pH values of 5 and 1.5. The cubes were submerged in these solutions for 120 days, and the pH values were monitored and kept constant. After a 120-day immersion, the TBC samples were removed from the solutions and allowed to dry for 24 h at room temperature. Thus, TBC’s resistance to sulfate and acidic attacks was examined by determining the residual density and compressive strength of the cubes in comparison with cubes immersed in water [20,51].

**(b) Drying shrinkage**

The linear drying shrinkage of TBC was determined per ASTM C426 [52] on 75 × 75 × 250 mm prism specimens with three replicates per specimen. A vertical comparator was used to monitor the samples’ length changes at predetermined intervals following demoulding after 24 h. All samples were exposed and dried in controlled conditions (23 ± 2 °C and 50 ± 3% RH) during the test duration. Thus, linear drying shrinkage at any time x (S_x_, %) was determined using Eq (1):


$$S_{x}=\frac{{\Delta L}_{x}}{G}$$ (1)

where ∆L_x_ represents the change in the linear dimension of the specimen due to drying from a saturated condition to the length of the specimen at any time, x (mm), and G is the test specimen gauge length (mm).

### 2.3. Characterizations of concrete samples

#### 2.3.1. Microstructures and elemental compositions.

The microstructures and elemental compositions of the concrete samples were examined using a scanning electron microscope (SEM) and energy dispersive X-ray spectroscopy (EDX). A platinum coating of electrically conducting material was applied to the crushed concrete samples using a low-vacuum sputter coating technique. The coated sample was subsequently examined with a field emission scanning electron microscope in secondary electron imaging mode [53]. EDX was employed to examine the distribution of elemental composition on a particular scale. Data for a small accelerating voltage of 20 kV with a one-minute scan time were acquired utilizing the EDX technique [54].

#### 2.3.2. Mineralogical identification.

The powdered samples were sieved to 0.074 mm after being pelletized. After that, the sieved samples were covered with paper and set on a flat glass plate inside a 35 mm × 50 mm aluminum alloy grid. Each sample was passed through a Rigaku D/Max-lllC X-ray diffractometer (Rigaku Int. Corp. Tokyo, Japan) with a CuKa radiation set at 40 kV and 20 mA. The diffractometer was configured to produce diffractions by a scanning rate of 1 ^o^/minute from 0–70 ^o^ at a scanning angle (2θ).

#### 2.3.3. Thermal analysis.

A simultaneous thermal analysis was used to evaluate the prepared samples’ thermal decomposition, phase transitions, melting point, and heat flow. After 28 days of curing, about 11.50 mg of the powdered samples were prepared, heated from 30–950 °C at a rate of 10 °C per minute, and analyzed for thermogravimetric analysis (TGA)/differential thermal analysis (DTA) using PerkinElmer instrument. For differential scanning calorimetry (DSC), approximately 5.50 mg of the powdered specimens were prepared, heated from 30–300 °C at a rate of 10 °C per min, and analyzed using Mettler Star SW 13.00.

## 3. Results and discussion

### 3.1. Workability

**Fig 5:**
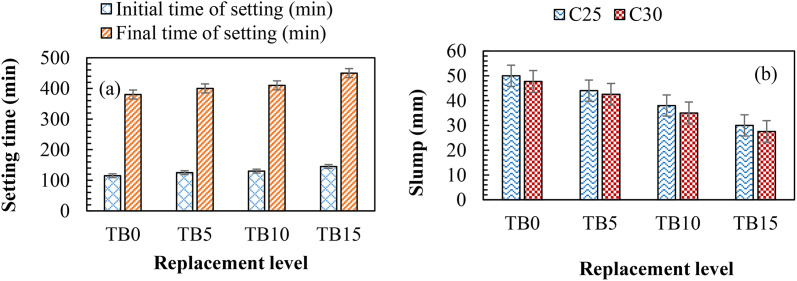
Workability showing (a) setting times of mortar samples and (b) slump values of fresh TBC samples.

Fig 5 (a) shows the setting times for TB0, TB5, TB10, and TB15 samples. The results revealed increased initial and final setting times with increasing SNA and GOS contents in the mixes. The initial setting times increased from 125 min at 5 wt. % SNA and GOS to 145 min at 15 wt. % SNA and GOS compared to the control specimen with 115 min. Similarly, as the SNA and GOS blends increased from 5 to 15 wt. %, the final setting times increased from 400 to 450 min compared to the control sample with 380 min. The increase in setting times is because ternary blends react with water more slowly. SNA and GOS’s diluting effects in the cement matrix might delay setting periods. SNA has less calcium content than GOS; this diminishes the SNA’s hydraulic reactivity, lengthening setting periods. A longer setting time could result from the increased amount of water needed for the mix and the initially delayed hydration by the ternary mix’s composition compared to utilizing cement [55]. These results corroborate pertinent studies that reported increased setting times with increased cement replacement with Oyster seashell ash [20,56] and shea nutshell ash [18]. Ultimately, in hot climates where extending the time available for normal concrete casting is advantageous, SNA and GOS can be used with cement.

As displayed in Fig 5 (b), Slump test findings revealed that workability decreases as cement replacement with SNA and GOS increases. The workability dropped from 50 mm to 30 mm for C25 and from 47.70 mm to 27.50 mm for C30 when the replacement level of SNA and GOS increased from 0 to 15 wt. % each. This can be explained by SNA and GOS having larger surface areas, as shown in Table 1, which raises water demand and makes fresh concrete stiffer and less workable. The main chemical component of the GOS is CaO; during the CaO slaking process, Ca(OH)_2_ is produced, and water is rapidly consumed. As a result, the slump in fresh concrete decreases. These are supported by previous research, which reported a reduction in workability with increasing Oyster seashell ash [57] and shea nutshell ash [18,58] in the blended mixes. Seashell wastes typically lower slump values of concrete due to their uneven shape and internal cavities, which increase particle friction and raise the water demand and absorption rate [19]. However, relevant studies found that the slump increased as the amount of seashell ash powder rose, most likely because it takes longer for seashell cement to hydrate than cement [20,59]. The practical implication is that TBC becomes stiffer with the addition of SNA and GOS, which increases labour demand and necessitates careful consolidation to prevent defects. Notwithstanding the challenges, low-slump concrete is purposefully utilized for applications requiring high strength and minimal permeability, including pavements, foundations, and road construction, where the concrete must maintain its shape.

### 3.2. Mechanical properties

#### 3.2.1. Compressive strength.

**Fig 6:**
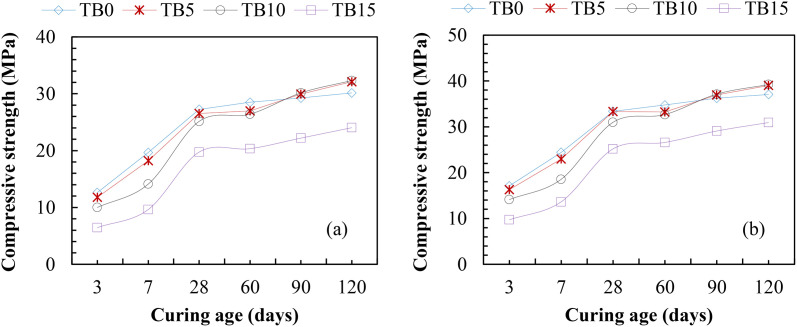
Compressive strengths of TBC for (a) C25 and (b) C30 MPa.

Compressive strength is the most crucial of concrete’s mechanical characteristics for assessing its performance under various loads. According to Fig 6, the early age compressive strength decreased as SNA and GOS increased in the blended mixes from 5–10 wt. %; however, the concrete’s compressive strength increased and eventually exceeded that of the control concrete at a later age. The reaction of CaO in GOS with gypsum and Al_2_O_3_ can prevent alite from hydrating early and reduce its compressive strength at an early age [20,60]. Gypsum generally accelerates early hydration but may interact with other components in the mixture, resulting in reduced early strength due to a lower percentage of effective reactions over time. The pozzolanic reaction between SNA and GOS, reactive calcite in GOS, reactive silica and alumina in SNA, and calcium ions in PLC, which create calcium silicate hydrate (C-S-H) and calcium silicate aluminate hydrate (C-S-A-H), are responsible for the improvement in compressive strengths at later ages. This strengthens the concrete by generating binding forces within the matrix [61]. For C25 in Fig 6 (a), as the SNA and GOS blend increased from 5–10 wt. %, the compressive strength, up to 60 curing age, was about 27 MPa compared to the TB0 (control concrete) with about 29 MPa, resulting in about 7% decrease in strength. However, at 120 curing age, the compressive strength increased to about 33 MPa at 5–10 wt. % SNA and GOS substitution compared to the control sample, with about 31 MPa, leading to about a 7% increase in strength. Like C25, C30 in Fig 6 (b) exhibited about 34 MPa at 60 curing age as the SNA and GOS blend increased from 5–10 wt. % compared to the control specimen with 35 MPa, indicating a 3% decrease in strength. Meanwhile, at a later age (120 curing age), TBC yielded a compressive strength of approximately 40 MPa at 5–10 wt. % SNA and GOS addition compared to the control concrete with 37.05 MPa, resulting in a 6% increase in strength. These results are consistent with pertinent research that found that adding SNA [18,58] and seashell ash powder [20,56,62] to blended cement concrete lowers the concrete’s early strength. Compared to the control sample, the compressive strength of blended cement concrete was modified by 10–20 wt. % SNA decreased by roughly 22–45% and 13–23% at 7–28 curing ages, but increased by 11–21% at 90 curing age [58]. Similarly, the compressive strength of a 5 wt. % seashell powder-based-blended cement concrete decreased by about 2% at 3–7 curing ages but increased by 2–3% at 28–90 curing ages compared to the control concrete [20].

The compressive strength of TBC declined at early and later ages at 15 wt. % GOS and SNA, as shown in Fig 6. At 28, 60, and 120 curing ages, the compressive strengths for C25, as indicated in Fig 6(a), dropped by roughly 38, 29, and 26% in comparison to TB0. Likewise, compared to the control concrete, there were about 33, 24, and 25% decreases in compressive strengths at 28, 60, and 120 curing ages for C30 in Fig 6 (b). The possible solubility of the carbonate compound upon its interaction with water during the curing process can be attributed to the decrease in compressive strength at TB15 mixtures (15 wt. % SNA and GOS substitution) [20]. Besides, the poor pozzolanic reaction between SNA and GOS can be responsible for the decreased compressive strength of TB15 mixes. SNA and GOS act more as fillers than binders within the cement paste matrix. As the replacement percentage increases, the surface area of fillers to be bonded by cement increases, resulting in poor matrix bonding and reduced strength. Related studies support these findings, where the 5–10 wt. % of Oyster seashell powder addition increased the concrete’s compressive strength by 7–14% compared to the control concrete [57]. However, adding 15–20 wt. % seashell powder reduces the concrete’s compressive strength by 11–16% and 7–15% at 28 and 90 curing ages. Ultimately, this study infers that 10 wt. % SNA and 10% GOS are the optimum replacement levels with PLC in TBC production, satisfying the required strength for building structural elements (foundations, footings, slabs, beams, and columns) and pavements.

#### 3.2.2. Flexural strength.

**Fig 7:**
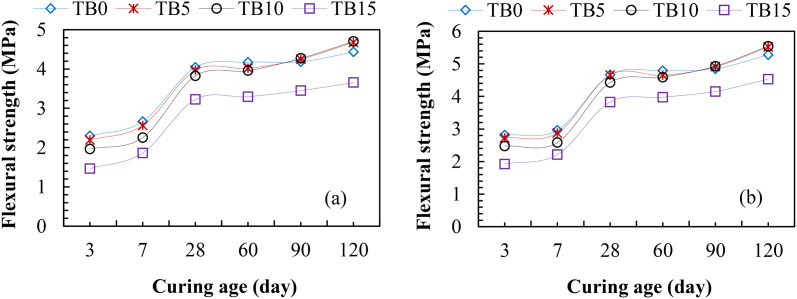
Flexural strengths of TBC for (a) C25 and (b) C30 MPa.

Fig 7 shows the flexural strengths of TBC samples for C25 and C30. The results exhibited a similar pattern to compressive strengths in Fig 7, increasing gradually with increased SNA and GOS contents up to 10 wt. % and decreasing at 15 wt. % for C25 and C30. From Figs 7 (a) and (b), the TBC mixes yielded lower flexural strengths at 3–60 curing ages than the TBC mixes. The flexural strengths, as shown in Fig 7 (a), varied from 2.19–4.02 MPa for TB5, 1.96–3.96 MPa for TB10, and 1.47–3.30 MPa for TB15, compared to the control mixes (TB0) with 2.30–4.17 MPa after 3–60 curing ages for C25. These resulted in about 4–5%, 5–15%, and 20–36% decreases in flexural strengths. Similar trends exist in Fig 7 (b) for C30 at 3–60 curing ages: TB5, TB10, and TB15 varied from 2.72–4.65 MPa, 2.48–4.59 MPa, and 1.93–3.98 MPa compared to the control mixes (TB0) with 2.82–4.79 MPa, leading to approximately 2–3%, 5–12%, and 17–32% strength reductions. Thus, the TBC mixes with 5–15 wt. % SNA and GOS dosages possessed relatively lower early age flexural strengths than the control mixes. The presence of SNA [18] and Oyster seashell powder [57] has been reported to exhibit a lower flexural strength performance at early ages compared to the control mixes due to the slow generation of pozzolanic reactivity between SNA and GOS compared to the primary hydration of PLC. However, from 90 curing age, the ternary blended concrete, at 5–10 wt. % SNA and GOS replacement levels exhibited higher flexural strengths than the control mixes. Nonetheless, beyond 10 wt. % replacement level, the presence of SNA and GOS contents does not improve the flexural strength of TBC. At 90 and 120 curing ages in Fig 7 (a), the flexural strengths ranged between 4.25 and 4.68 MPa and 4.27 and 4.70 MPa for TB5 and TB10, compared to the TB0, with 4.18 and 4.43 MPa. These led to about a 2–6% strength increase. Similar results were obtained by TB5 and TB10 in Fig 7 (b), resulting in flexural strengths of 4.91 and 5.44 MPa and 4.93 and 5.54 MPa, compared to TB0, with 4.85 and 5.28 MPa at 90 and 120 curing ages. These led to an increase in flexural strengths of between 1–3% and 2–5%. The increase in flexural strengths at a later age can be attributed to the presence of SNA and GOS, which generate the secondary C-S-H and C-A-S-H hydrates. These fill up the voids in the primary C-S-H network formed by the primary hydration of PLC, interlocking the particles in the cementitious matrix and resulting in increased strengths. These results support the previous findings where the flexural strengths of 5–10 wt. % Oyster seashell powder-based-cement concrete varied between 9.3 and 9.8 MPa [57] and 7.12 and 8.92 MPa [63] compared to the control mixes with 8.5 and 5.84 MPa at later ages. Ultimately, this study recommends a 10 wt. % SNA and GOS dosage as an optimum replacement level in the production of ternary blended concrete to resist bending or deformation under an applied load.

#### 3.2.3 Split tensile strength.

**Fig 8:**
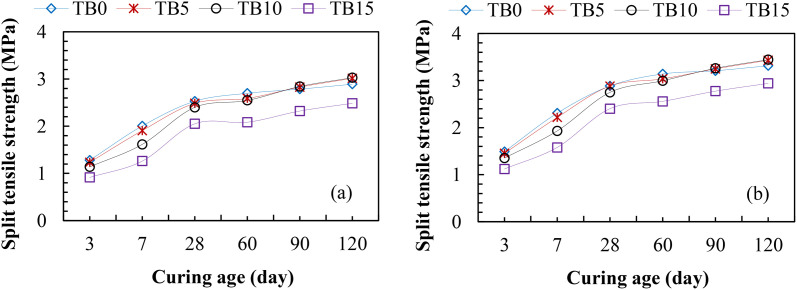
Split tensile strengths of TBC for (a) C25 and (b) C30.

Similar to compressive and flexural strengths in Figs 6 and 7, the results of split tensile strengths for TBC are presented in Fig 8. The results revealed lower split tensile strengths at 3–60 curing ages than the control samples due to the slow pozzolanic reactivity of SNA and GOS compared to the primary hydration of PLC. For C25 in Fig 8 (a), the split tensile strengths of TB5, TB10, and TB15 varied from 1.24–2.59 MPa, 1.14–2.55 MPa, and 0.91–2.08 MPa, compared to TB0 with 1.28–2.70 MPa after 3–60 curing ages. Similarly, the results of split tensile strengths in Fig 8 (b) for C30 varied from 1.45–3.14 MPa, 1.35–2.99 MPa, and 1.12–2.56 MPa for TB5, TB10, and TB 15 compared to TB0 with 1.12–2.56 MPa. However, due to the good interlocking between the binder matrix and aggregates, 5 and 10 wt. % SNA and GOS’s replacement levels, as shown in Fig 8 (a) and (b) for C25 and C30, yielded higher split tensile strengths than control mixes. For C25, the split tensile strengths were 2.83 and 3.01 MPa and 2.82 and 3.03 MPa for TB5 and TB10, compared to TB0 with 2.79 and 2.89 MPa, signifying between 1 and 5% increases in split-tensile strength. Similarly, TB5 and TB10’s spilt tensile strengths were 3.25 and 3.43 MPa, and 3.26 and 3.44 MPa, compared to TB0 with 3.21 and 3.3 MPa for C30. These results are in consonance with the split tensile strengths 3.35, 3.42, 2.64, and 2.25 MPa for 5, 10, 15, and 20 wt. % bivalve clam seashell powder-based-cement concrete compared to the control sample with 2.98 MPa [20]. These signify about an 11–13% increase and a 12–15% decrease in split tensile strength at 5–10 wt. % and 15–20 wt. % replacement levels. Therefore, a 10 wt. % SNA and GOS blend with PLC is recommended for TBC, satisfying the ability to withstand a split or fracture under an applied load.

### 3.3. Strength modelling and validation

#### 3.3.1. Modelling.

**Fig 9:**
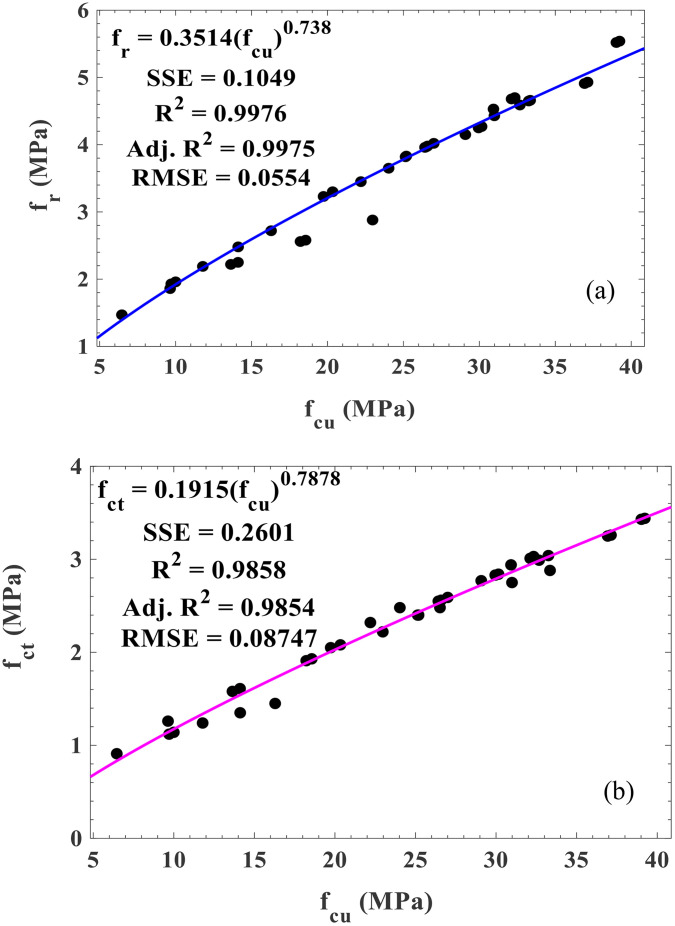
Relationship between (a) flexural and compressive strengths and (b) split tensile and compressive strengths of TBC.

The relationship between the flexural and compressive strengths and split tensile and compressive strengths are presented in Figs 9 (a) and (b). From Fig 9 (a), the modelling is 99.76% at a 95% confidence bound fit to predict the flexural strength from compressive strengths. Besides, other performance metrics, SSE (0.1049) and RMSE (0.0554), are closer to zero, indicating a strong correlation. This model aligns with the 97.20% R^2^ obtained for predicting the relationship between the flexural and compressive strengths of concrete [49]. In the same vein, Fig 9 (b) generates a regression equation with 98.58 R^2^ at 95% confidence bound to predict the relationship between the split tensile and compressive strengths of TBC modified with SNA and GOS. In addition, the SSE and RMSE values of 0.2601 and 0.08747 are closer to zero, signifying a strong correlation [64]. This model corroborates the 96.80% R^2^ generated from forecasting the relationship between the split tensile and compressive strengths [49]. Hence, the proposed models in Figs 9 (a) and (b) are beneficial in predicting the flexural and split tensile strengths of TBC modified with SNA and GOS from compressive strengths ranging from 7–40 MPa at 3–120 curing ages, minimizing time and materials for conducting experimental works.

#### 3.3.2 Model validation.

**Fig 10:**
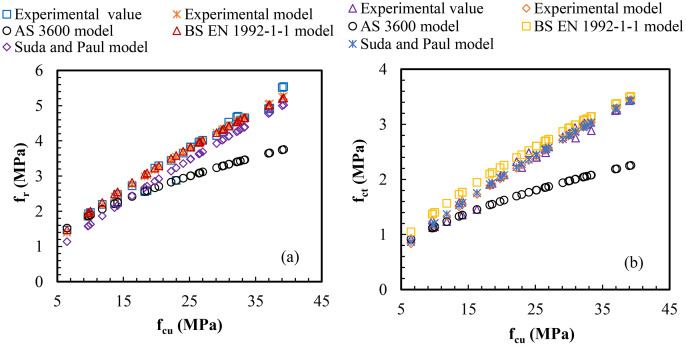
Model validation between (a) flexural and compressive strengths and (b) split tensile and compressive strengths of TBC.

The models developed in Figs 9 (a) and (b) were validated with existing models, and the results are indicated in Fig 10. From Fig 10 (a), as the compressive strengths of all TBC mixes increased, the flexural strengths increased in a similar trend, with experimental value and model resulting in strong alignment. The BS EN model [49] is strongly close to the data points of experimental value and model of compressive strengths at early and later ages. Moreover, Suda and Paul’s model [50] is nearly located at the experimental value and model at the later ages of compressive strength; however, there is a little gap at the early ages of compressive strength. AS 3600’s model [48] is close to the data points of experimental value and model at the lower range of compressive strength (up to 15 MPa); beyond this, the model predicted a lower flexural strength at higher compressive strength ranges. Thus, it can be asserted that the BS EN model exhibited the best validation for predicting the flexural strength of SNA-GOS-PLC-based concrete at the early and later ages of compressive strength.

The trends shown in Figs 10 (b) and (a) are identical, meaning that for all TBC mixtures, higher compressive strengths resulted in higher flexural strengths. From Fig 10 (b), the experimental value and model are closely aligned. The BS EN model [49] is located near the regression points of the experimental value and model. It marginally predicted higher split tensile strengths at early and later ages of compressive strengths. Interestingly, unlike Fig 10 (a) validation, Suda and Paul’s model [50] is closely located at the experimental values and model’s regression points, forecasting the flexural strengths at early and later ages of compressive strengths. AS 3600’s model [48] is near the experimental value and model data points at early compressive strength ages (up to 15 MPa); after that, the model projected a lower flexural strength at later compressive strength ages. Suda and Paul’s model provided the best validation for predicting the split tensile strength of SNA-GOS-PLC-based concrete in the early and later ages of compressive strength. The types of mixes, the water-to-binder ratio, and the physical and chemical compositions of the binding agents used to produce the concrete mixes could all be responsible for the variations in the validation, particularly the AS 3600 model [65].

### 3.4. Durability properties

#### 3.4.1. Acidic resistance.

**Fig 11:**
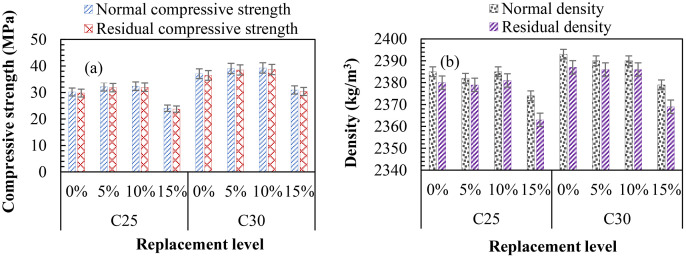
Acidic resistance indicating (a) residual compressive strength and (b) residual density.

Fig 11 shows the acidic attack test results. The residual strength and density for each mix are evaluated in consonance with samples cured in normal water for 120 days. From Fig 11, the TBC incorporating 5–10 wt. % SNA and GOS showed comparatively lower strength and density changes than the control sample. The percentage strength losses after 120 days of immersion in 5% H_2_SO_4_ solution were 1 and 1.20% for TB5 and TB10, compared to TB0, with 1.35% for C25, and 0.77 and 0.89% for TB5 and TB10, compared to TB0, with 1.05% for C30 in Fig 11 (a). Similarly, there were 0.13 and 0.17% density losses after 120 days of immersion in 5% H_2_SO_4_ solution for TB5 and TB10, compared to TB0 with 0.21% for C25. For C30, a 0.17% density loss was recorded for TB5 and TB10 compared to TB0, with 0.25% in Fig 11 (b). These outcomes are explained by SNA and GOS creating stronger gels with less Portlandite (Ca(OH)_2_); hence, adding SNA and GOS to PLC increases TBC tolerance in acidic environments. Beyond certain threshold values, calcium concentrations increase the concrete’s capacity to absorb sulfuric acid [66]. Adding SNA and GOS to concrete reduces capillary pores and improves hydration, enhancing C-S-H gel formation and less acidation on TBC. These findings support earlier studies that showed that adding pozzolans and SCMs to blended cement concrete increases its tolerance to acidic environments [67,68]. For instance, TBC made with 8% silica fume and 20% fly ash in addition to OPC demonstrated a notable resistance to acidity compared to binary mixed concrete and OPC-based concrete [69]. Moreover, after 25 h of submersing PC concrete and 35 wt. % RHA-based-PC concrete in 5% H_2_SO_4_ solution, the weight losses were 27 and 13% [68]. OPC concrete exhibits poor resilience to acidic environments because it contains 60–65% CaO, and its hydration products comprise around 25% Ca(OH)_2_. In contrast, RHA-based-PC concrete contains 20–40% CaO and almost no Ca(OH)_2_ as hydration products [68]. In comparison to PC concrete, a 10 wt. % metakaolin-based PC concrete showed enhanced service life and a notable resistance to acidic attack [67].

Nonetheless, TB15 exhibited higher strength and density changes than TB0 after 120 days of immersion in 5% H_2_SO_4_ solution, as shown in Fig 11. The strength losses were 1.38 and 1.15% for C25 and C30, compared to TB0, with 1.35 and 1.05%. Likewise, the density losses were 0.51 and 0.25% compared to TB0 with 0.21 and 0.17%. These outcomes are explained by the acid medium’s attack on cement matrix hydration products, which induces hydrolytic disintegration by eroding the density and strength of TB0. Another study stated that the 5% H_2_SO_4_ solution has a significant impact on 10 wt. % green gram pod ash-based cement concrete compared to the control mix was because of the reaction between acid and concrete, which was more noticeable in the presence of calcium-containing fillers [70]. This reaction generated calcium sulfates, especially water-soluble salts, which reduced the concrete’s strength and weight. Thus, at their optimal levels of 10%, SNA and GOS can react with the acid around the concrete, reducing the vulnerability of TBC to acidic damage.

#### 3.4.2. Sulfate resistance.

**Fig 12:**
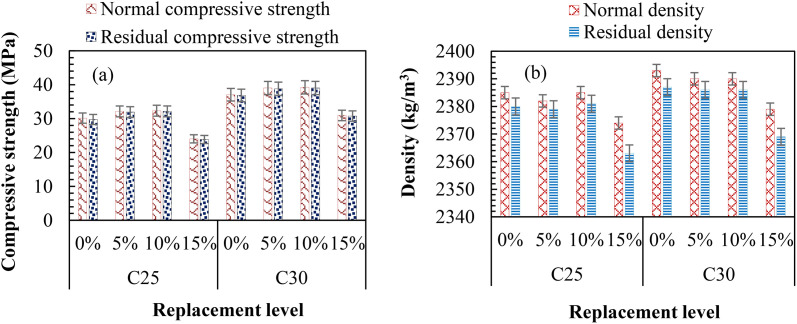
Sulfate resistance indicating (a) residual compressive strength and (b) residual density.

Fig 12 (a) and (b) indicate the difference in strength and density loss due to the sulfate resistance test after 120 days of immersion in a 5% MgSO_4_ solution. As shown in Fig 12, the results revealed a decreased sulfate attack with increased SNA and GOS dosages in the TBC mixes up to 10 wt. %. Beyond a 10 wt. % replacement level, the resistance to sulfate attack declined. From Fig 12 (a), TB5 and TB10 showed strength losses of 0.61 and 0.62%, compared to TB0, with 0.70% for C25, and 0.50 and 0.51%, compared to TB0, with 0.57% for C30. Similarly, Fig 12 (b) indicates a density loss of 0.08% for TB5 and TB10 compared to TB with 0.13% for C25. For C30, TB5 and TB10 exhibited density losses of 0.08 and 0.13% compared to 0.17% for TB0. Ettringite is linked to the sulfate-induced expansion of concrete, making it more vulnerable to a magnesium sulfate attack [66]. Additionally, the development of expansive gypsum and ettringite may result in a sulfate attack, causing the concrete to deteriorate, crack, spall, and expand. Gypsum and ettringite are the outcomes of the sulfate’s reaction with various OPC paste products [65]. Because of the calcium compounds that are formed and deposited on the surface of the TBC, TB5, and TB10 are more resistant to sulfate attack than TBO. As a result, ettringite cannot develop when sulfates are present, reducing permeability and preventing dangerous sulfate ions from entering the TBC [71]. These results align with Tayeh et al. [20]’s, which found that after 60 days of immersion in 5% MgSO_4_ solutions, the percentage weight and compressive strength losses for 5 wt. % seashell powder-based cement concrete were 0.62 and 1.09% compared to the control mixes with 0.65 and 1.17%. Furthermore, sulfate attack on the concrete cubes increased slightly due to the nutshell particles’ high filling capacity and impermeable character: SNA increased sulfate attack by 21% at the 30% replacement compared to the control concrete after 90 days of immersion in 5% MgSO_4_ solution [18]. In similar findings, mortar specimens’ expansion and compressive strength loss decreased with the increase in percentage replacement of OPC with black rice husk ash after 180 days of immersion in 5% MgSO_4_ solution [72]. In addition, concrete made with RHA and OPC demonstrates superior sulfate resistance compared to blended SF and OPC [67].

However, the MgSO_4_ solution had a more significant influence on the TB15 mix than the other mixes: sulfate resistance decreased with excessive SNA and GOS dosages in the TBC. The results in Figs 12 (a) and (b) indicated the strength and density losses of 1.38 and 0.21% for C25 and 0.61 and 0.21% for C30. These results can be credited to the infiltration of sulfate ions (SO_4_^2-^), which cause gypsum formation within the microstructure, resulting in ettringite formation and expansion [20,73]. A similar study reported percentage weight and compressive strength losses of 0.77 and 1.14% for 15 wt. % and 0.69 and 1.59% for 20 wt. % seashell powder-based-cement concrete compared to the control mix with 0.65 and 1.17% after 90 days of immersion in 5% MgSO_4_ solution [20]. Mehta [68] also showed that the OPC paste boosts the ettringite’s water absorption values and reduces stiffness when submerged in sulfate solutions. Additionally, the expansion and cracking brought on by sulfate attack decrease the cohesiveness of the hydrated OPC paste and the adhesion between the aggregate particles, which raises the compressive strength loss of concrete specimens [67]. Hence, the inclusion of SNA and GOS at 10 wt. % optimum for TBC production offers a better resistance to sulfate attack. This can be applied in an environment prone to sulfate attack.

#### 3.4.3. Drying shrinkage.

**Fig 13:**
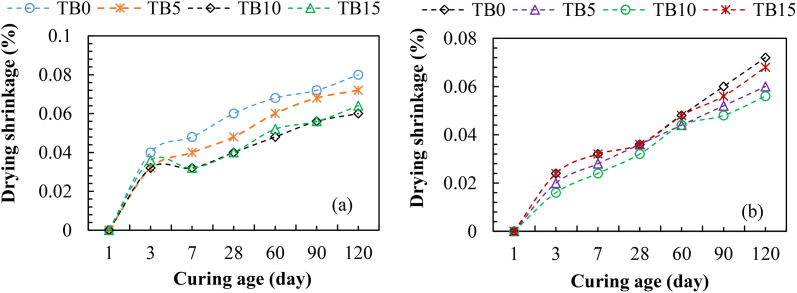
Drying shrinkage for (a) C25 and (b) C30.

Drying shrinkage has a major effect on the durability (long-term) performance of concrete structures, mainly by causing internal stresses within the concrete as it dries, resulting in cracking, water penetration, chemical attacks, and other forms of deterioration rather than directly affecting the concrete’s mechanical strength under load. Hence, Fig 13 compares the drying shrinkage of TBC samples to the control samples after 1–120 curing ages. Fig 13 shows that the drying shrinkage had no impact on day 1. After day 1, the drying shrinkage increased with increased curing ages due to cementitious hydration. Interestingly, the drying shrinkage decreased as the SNA and POS contents increased in the mixes. The drying shrinkages of TB5, TB10, and TB15 were 6–20%, 20–34%, and 10–34% smaller than the control sample (TB0) as the curing ages rose from 3–120 in Fig 13 (a). Similarly, in Fig 13 (b), TB5, TB10, and TB15 showed drying shrinkages that were 9–17%, 9–34%, and 0–7% lower than the control specimen (TB0). The participation of SNA and GOS particles in the hydration process is responsible for the decreased drying shrinkage; as the volume increases and the surrounding cement hydration products are squeezed, the concrete comparatively loses C-S-H change, improving the compactness of the cementitious-aggregate body structure. The amount of C-S-H gel rises as more cement particles hydrate, creating a greater network of linked pores that are more likely to lose water during drying and cause noticeable shrinkage in control samples compared to TBC samples [74]. Various studies reported the effects of pozzolans and SCMs on the drying shrinkage of concrete. For instance, incorporating silica fume (SF) and granulated blast furnace slag (GBFS) decreases the drying shrinkage of concrete [74–76]. The partial replacement of PC with 10–20 wt. % gravel wash mud powder reduced the concrete’s drying shrinkage by 4–8% [77]. However, the incorporation of fly ash exceeding 30 wt. % weakened the shrinkage’s reduction effect on concrete [74], while up to 20 wt. % substitution showed no discernible differences between the fly ash and non-fly ash-based concrete [78]. Other studies reported that silica fume increased the drying shrinkage of concrete [75,76]. These diverse impacts might result from different materials’ physical and chemical properties. The drying shrinkage for C25 in Fig 13 (a) was more than that for C30 in Fig 13 (b). This is because a higher water-to-binder ratio increases water availability for hydration, which raises the degree of hydration and results in greater drying shrinkage. Ultimately, a 10 wt. % SNA and GOS induced the best reduction of drying shrinkage compared to other TBC samples.

### 3.5 Characterizations

#### 3.5.1 SEM-EDX

**Fig 14:**
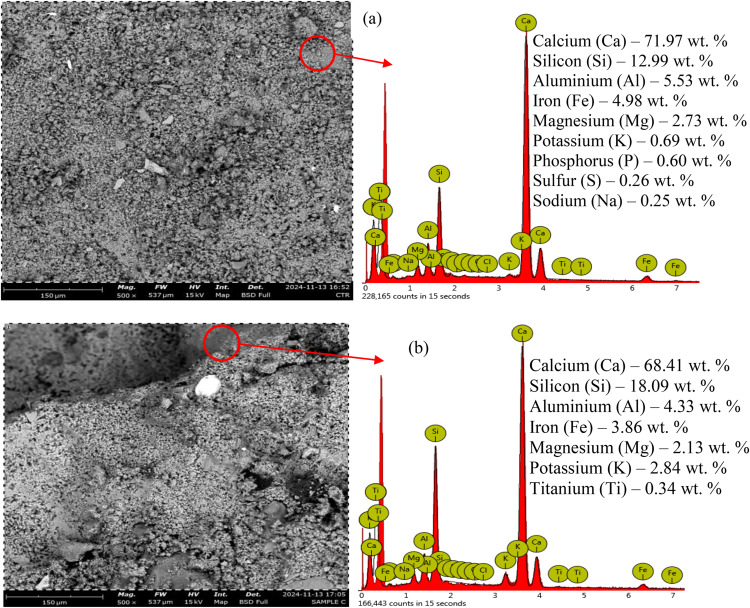
Morphology of (a) control sample (TB0) and (b) TB10 after 28 curing age.

Fig 14 displays the SEM-EDX of control concrete (TB0) and 10 wt. % SNA-GOS-based TBC (TB10). From Figs 14 (a) and (b), TB0 and TB10 revealed dense and compact structures. However, the inclusion of 10 wt. % SNA and GOS dosages in Fig 14 (b) introduce amorphous C-S-H gel, microscopically tiny protuberances, and irregularities. The EDX analysis highlights the compositional differences between TB0 and TB10. The replacement of the PLC with 10 wt. % SNA and GOS altered the elemental components of concrete and introduced new elements. The EDX plots of TB0 and TB10 in Figs 14 (a) and (b) showed calcium (Ca) and silicon (Si) as peak elements at 3.75 and 1.75 keV. The most significant change is the marginal decrease in calcium content, from 71.97 wt. % in TB0 to 68.41 wt. % in TB10. Calcium is essential for cement hydration, which increases concrete’s strength and durability. Aluminium content marginally reduced from 5.53 wt. % in TB0 to 4.33 wt. % in TB10. Aluminium influences concrete’s hydration process and long-term performance. Nonetheless, Silicon content increased to 18.09 wt. % in TB10 from 12.99 wt. % in TB0. Silicon is crucial to concrete because it increases its strength, resilience to environmental influences, and durability. The decrease in Ca and Al contents was compensated by an increase in Si content, leading to high mechanical and durability properties of TB10 compared to TB0. Therefore, adding SNA and GOS to the synthesis of TBC results in a new hydrated cement matrix with improved cohesion and performance. According to similar research, adding the seashell powder to cement mixtures causes more ettringite- and calcium-carbo aluminate-like phases, which tend to rise as the amount of seashell powder in the mix increases [53,56]. Some ettringite and calcium carbo-aluminate are found to form near or on the seashell powder location. This explains why seashell powder enriches the hydrated cement matrix (forming a composited matrix) and promotes (acting as nucleation sites) the precipitation of hydration products, particularly ettringite and calcium carboaluminate [56].

#### 3.5.2. XRD

**Fig 15:**
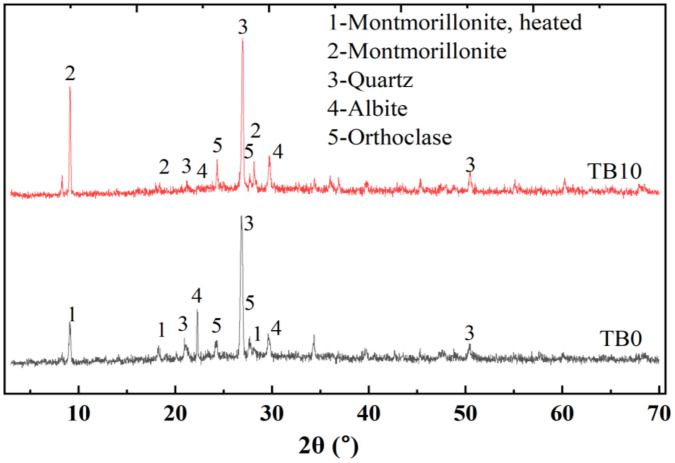
XRD spectrum of TB0 and TB10.

The XRD patterns of TB0 and TB10 are shown in Fig 15. The main hydrated phases are montmorillonite, quartz, albite, and orthoclase. The major hydrated peaks are montmorillonite and quartz for TB0 and TB10 at 9 and 27^o^ of diffraction angular range 2θ. Interestingly, including SNA and GOS increases the diffraction peak of montmorillonite for the TB10 mix. Montmorillonite contains mostly SiO_2_ in its chemical composition. The silica (SiO_2_) reacts with Ca(OH)_2_ in PLC, generating C-S-H [79]. These hydrates are responsible for the development of the strength of the hydrated cement paste. For example, the filler effect, dispersion, and pozzolanic reaction of montmorillonite with Ca(OH)_2_ led to about a 12% increase in compressive strength of blended cement mortar compared to the mortar sample without montmorillonite [79]. Thus, the XRD pattern results were consistent with the results of the aforementioned mechanical properties.

#### 3.5.3. TGA/DTA

**Fig 16:**
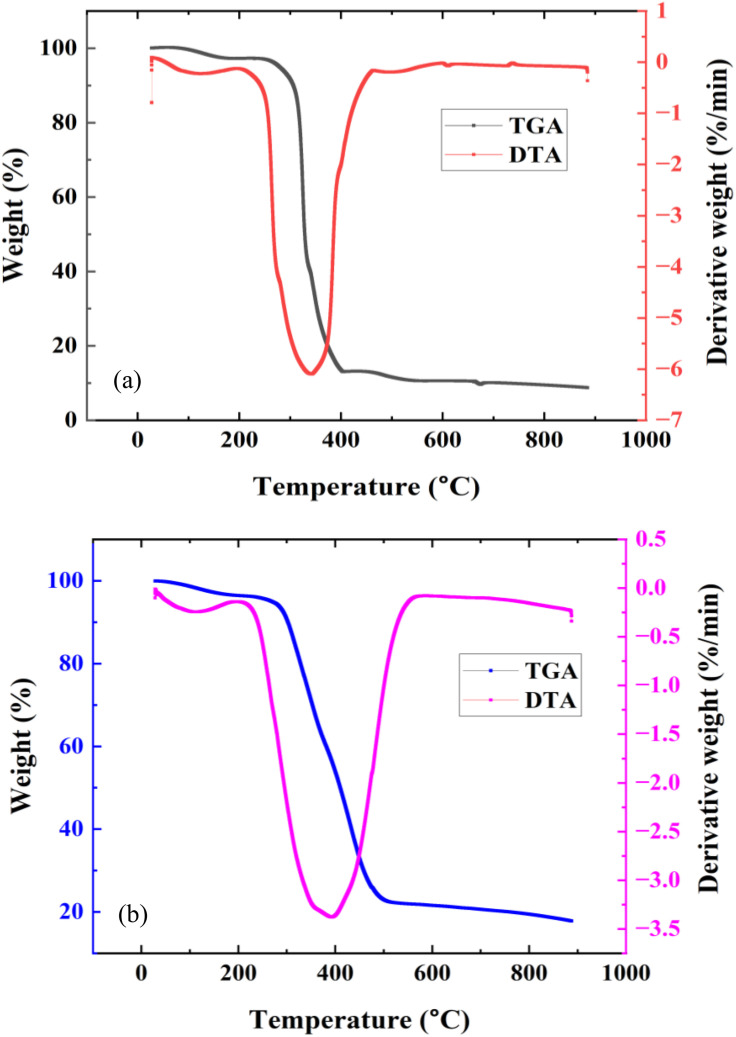
TGA/DTA curves for (a) control sample (TB0) and (b) TB10 after 28 curing ages.

Fig 16 displays the findings from the TGA/DTA tests conducted on TB0 and TB10. The DTA profiles indicated the normal reactions in the samples when exposed to incremental temperatures (30–950 °C) after 28 days of curing age. From Fig 16 (a), the Dehydroxylation of the C-S-H phase and ettringite decomposition are responsible for the first endothermic peak, corresponding to the mass loss on the TGA profile up to 300 ◦C. The primary cause of this stage’s weight loss is the weakly bound water physically absorbed from the gel solid at 110–120 °C [80]. The second major peak is equally endothermic, with a net mass loss beginning at roughly 425 ◦C due to the release of crystallized water from the dehydration of Ca(OH)_2_ [80,81]. The CaCO_3_ decarbonization due to lime formation [82] represents the final endothermic peak between 550 and 900 °C. The sample (TB0) lost 90% of its total weight. The degree of reactivity of the primary clinker compounds is indicated indirectly by this. Since one of the byproducts of the silicate reaction with water is the Ca(OH)_2_ phase, the growth of the second peak further supports the idea that more hydration took place [80]. These results are consistent with pertinent studies whose mass losses due to dehydroxylation of C-S-H, dehydration of Ca(OH)_2_, and decarbonization of CaCO_3_ ranged from 25–210 °C [80,81,83,84], 418–530 °C [80,81,83,85], and 450–850 °C [80,81,83,86].

Fig 16 (b) displays the TG/DTA thermal analysis of TB10. The curve indicated a stronger first peak compared to the TB0. The sample was less hydrated, as evidenced by the overall weight loss of almost 80%, which is less than the TB0 value. The second peak, related to the Ca(OH)_2_ formation, was marginally smaller than that of TB0. This is explained by the pozzolanic effect, which breaks down Ca(OH)_2_ and produces secondary C-S-H [83]. Adding 10 wt. % SNA and GOS contents in the blended mix cause a decline in the total Ca^2+^ content, as evident in Fig 14, and subsequently reduce the formation of Ca(OH)_2_. Hence, these outcomes can be attributable to the superior mechanical and durability performance of TBC compared to the control concrete.

#### 3.5.4. DSC

**Fig 17:**
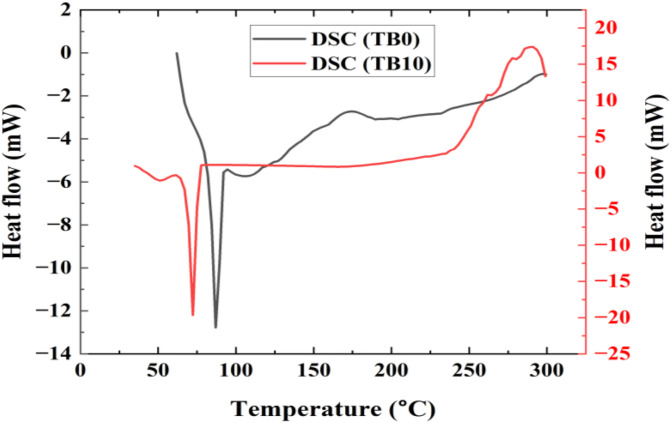
DSC curves for TB0 and TB10 after 28 curing ages.

Fig 17 illustrates DSC curves with phase transitions of TB0 and TB10 upon the first heating cycle. As evidence of the samples’ initial glassy condition, the small endothermal peaks at 75 and 62.50 °C correlate to the glass transitions of TB0 and TB10. At 87.50 and 70 °C, crystallization occurs exothermally in TB0 and TB10 after additional heating, followed by melting at 300 and 287.50 °C for TB0 and TB10. From Fig 17, the temperature decreased by 10 wt. % SNA and GOS contents in the mix. These results reveal the extent of the pozzolanic reaction in the investigated concretes, which liberate Ca(OH)_2_ and generate secondary C-S-H for strength development [87,88].

## 4. Conclusions

This research investigates the influence of 5–15 wt. % of SNA and GOS substitution on the mechanical properties of TBC after 3–120 curing ages. The durability performance was evaluated by testing the drying shrinkage of concrete specimens after 1–120 curing ages and the chemical resistance after 120 days of immersion in 5% H_2_SO_4_ and 5% MgSO_4_ solutions. The concrete samples were characterized for microstructures, elemental compositions, mineralogical properties, and thermal decompositions. The following conclusions were drawn based on the experimental findings:

a Initial and final setting times increased with increasing SNA and GOS contents in TBC mixes by 8–21% and 5–16% at 5–15 wt. % replacement levels compared to the control mixes. Besides, at 5–15 wt. % SNA and GOS replacement levels, the slump of TBC mixes reduced by 40–43% compared to the control mixes.
b The inclusion of 5–15 wt. % of SNA and GOS dosages resulted in decreased strengths. However, the later age strengths of TBC mixes at 5–10 wt. % outperformed the control mixes with 6–7%, 2–6%, and 1–5% increase in compressive, flexural, and split-tensile strengths after 90 curing days compared to the control concrete.
c At 90–120 curing ages, 5–10 wt. % of SNA and GOS substitution led to increased compressive, flexural, and split tensile strengths of 3–7%, 2–6%, and 2–5% for TBC mixes compared to the control mixes.
d The developed models yielded strong correlations with 99.75 and 98.54% R^2^ for the relationship between the flexural and compressive strengths and split tensile and compressive strengths.
e Incorporating 5–10 wt. % of SNA and GOS dosages improves the chemical resistance of TBC mixes with 11–27% and 19–40% resistance increases in compressive strength and density against acidic attack after 120 days of immersion compared to the control mixes. There were about 12% and 24–53% resistance increases in strength and density against sulfate attack compared to the control mixes.
f Drying shrinkage reduces with increasing SNA and POS contents in the TBC mix. A TB10 mix exhibited the optimum performance with 20–34% and 9–34% reductions in C25 and C30 compared to the control mix.
g Including SNA and GOS dosages in the blended cement concrete generates secondary C-S-H and enhanced montmorillonite for superior mechanical and durability performance.

This study fills knowledge gaps by demonstrating the viability of SNA and GOS as alternative cementitious blends for improved mechanical and durability performances of TBC. The research has reduced the cement content required to make structural and general-purpose concrete by 20 wt. %, facilitating sustainable production and consumption. Despite these promising results, further studies can investigate the effects of SNA and GOS on the modulus of elasticity, water permeability, chloride ion penetration, carbonation, and the economic feasibility of TBC.

## Abbreviations

C25: Grade 25 MPa concrete
C30: Grade 30 MPa concrete
CA: Coarse aggregates
C-A-S-H: Calcium aluminate silicate hydrate
C-S-H: Calcium silicate hydrate
DSC: Differential scanning calorimetry
DTA: Differential thermal analysis
EDX: Energy dispersive X-ray spectroscopy
F: Fine aggregates
f_ct_: Split tensile strength
f_cu_: Compressive strength
f_r_: Flexural strength
GOS: Ground oyster seashell
PLC: Portland limestone cement
SCMs: Supplementary cementitious materials
SEM: Scanning electron microscope
SNA: Shea nutshell ash
TBC: Ternary blended concrete
TGS: Thermogravimetric analysis
XRD: X-ray diffractometer
XRF: X-ray fluorescence
